# Angiopoietin 2 induces astrocyte apoptosis via *α*v*β*5-integrin signaling in diabetic retinopathy

**DOI:** 10.1038/cddis.2015.347

**Published:** 2016-02-18

**Authors:** J-H Yun, S W Park, J H Kim, Y-J Park, C-H Cho, J H Kim

**Affiliations:** 1 Department of Pharmacology and Ischemic/Hypoxic Disease Institute and Cancer Research Institute, Seoul National University College of Medicine, Seoul, Republic of Korea; 2 Cancer Research Institute, Seoul National University College of Medicine, Seoul, Republic of Korea; 3 Department of Biomedical Sciences, Seoul National University College of Medicine, Seoul 110-799, Republic of Korea; 4 Fight against Angiogenesis-Related Blindness (FARB) Laboratory, Biomedical Research Institute, Seoul National University Hospital, and Department of Biomedical Sciences and Ophthalmology, Seoul National University College of Medicine, Seoul, Republic of Korea; 5 Immunotherapy Research Center, Korea Research Institute of Bioscience and Biotechnology, Yuseong-gu, Daejeon, Republic of Korea; 6 Department of Ophthalmology, Seoul National University College of Medicine, Seoul, Republic of Korea

## Abstract

The vascular leakage in diabetic retinopathy leads to macular edema and vision loss. Although astrocyte play an important role in regulating blood-brain barrier integrity in the brain, the precise role of astrocyte in blood-retinal barrier was yet to be elucidated. This study aimed to investigate the role of angiopoietin 2 (Ang2) in astrocyte loss and vascular leakage in the early streptozotocin-induced diabetic retinopathy. We demonstrated that vascular leakage occurred with astrocyte loss in early diabetic mice retina as Ang2 increased. The astrocyte loss and vascular leakage were inhibited by intravitreal injection of Ang2-neutralizing antibody. *In vitro*, Ang2 aggravated high glucose-induced astrocyte apoptosis via GSK-3*β* activation. Ang2 directly bound to *α*v*β*5 integrin, which was abundant in astrocyte, and the blockade of *α*v*β*5 integrin, *in vitro*, effectively attenuated Ang2-induced astrocyte apoptosis. *In vivo*, intravitreal injection of anti-*α*v*β*5-integrin antibody inhibited astrocyte loss in early diabetic retinopathy. Taken together, Ang2 induced astrocyte apoptosis under high glucose via *α*v*β*5-integrin/GSK-3*β*/*β*-catenin pathway. Therefore, we suggest that Ang2/integrin signaling could be a potential therapeutic target to prevent the vascular leakage by astrocyte loss in early diabetic retinopathy.

Diabetic retinopathy (DR) is the most common microvascular complication and the leading cause of vision impairment in the diabetic patients and working-aged people.^1^ Although neovascularization cause severe vision loss only in the later proliferative phase of DR, macular edema caused by vascular leakage can occur at any stage of DR and impair visual acuity.^2^

In brain, the role of astrocyte as a component of neurovascular unit in the regulation of the blood-brain barrier (BBB) is well established. The perivascular endfeet of astrocyte contribute to the BBB features.^3^ In addition, astroglial-endothelial signaling regulates the BBB under pathological conditions.^3, 4^ Recently, retinal dysfunction in diabetes is explained by a subsequent change of the retinal neurovascular unit in terms of retinal pathophysiology.^1, 5^ However, the precise role of astrocyte in the regulation of the blood-retinal barrier (BRB) in diabetic retina has not been elucidated.

In the retina, both astrocyte and Müller cells are involved in vascular ensheathment. However, astrocyte and Müller cells are affected by diabetes in different ways.^6, 7^ Although astrocyte is limited to the ganglion cell layer, astrocyte plays an important role in vessel integrity both via direct contact and through humoral factors.^8, 9^ In addition, astrocyte is closely related to the formation of tight junction in developing retina.^10, 11, 12^ Moreover, retinal astrocyte may decrease in number at sites where vessel is damaged with increased permeability of the BRB in pathologic conditions.^6, 13, 14, 15^ Thus, in this study, we focused on the role of astrocyte regarding to the vascular leakage in diabetic retina.

Recently, we have demonstrated that angiopoietin 2 (Ang2) induces pericyte apoptosis via integrin signaling in DR.^16^ Hyperglycemia-induced Ang2 has a central role in pericyte apoptosis by Ang2/*α*3*β*1-integrin/p53 signaling pathway in streptozotocin (STZ)-induced diabetic mice. Local vascular leakage occurs early in the experimental diabetic mice even before the pericyte loss.^5^ In addition, Ang2 is upregulated by hyperglycemia in endothelial cells and diabetic retina.^17, 18, 19^ Moreover, Ang2 binds to integrin and regulates angiogenesis.^20^ In view of neurovascular unit, we postulated that Ang2 could affect astrocyte via integrin signaling before the pericyte loss in diabetic retina.

Here, we provide evidence for the role of Ang2-induced astrocyte loss in vascular leakage in early diabetic retina. We demonstrate that Ang2 shows synergistic effects with high glucose on astrocyte apoptosis via GSK-3*β*/*β*-catenin pathway. Furthermore, we demonstrate both *in vitro* and *in vivo* that blocking *α*v*β*5 integrin effectively attenuate Ang2-induced astrocyte apoptosis. Taken together, Ang2-induced astrocyte apoptosis via *α*v*β*5-integrin signaling contributes to the vascular leakage in diabetic retina.

## Results

### Retinal astrocyte loss occurs with retinal vascular leakage in early diabetic mice

**Figure 1:**
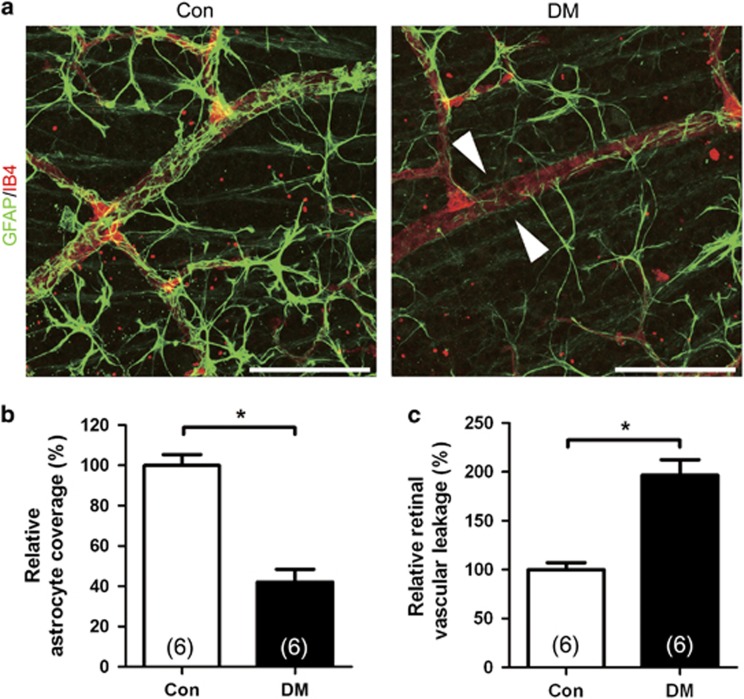
Retinal astrocyte loss occurs with retinal vascular leakage in early diabetic mice. Retinal vascular leakage and distribution of astrocyte were evaluated in 3-week streptozotocin-induced diabetic mice (DM) and age-matched controls (Con). (**a**) Representative site of focal astrocyte loss in mid-peripheral retina is shown in diabetic retina (original magnification × 400; scale bar, 100 *μ*m). White arrowheads indicate loss of astrocytes on diabetic retinal vessels. (**b**) Relative astrocyte coverage (colocalized area of astrocyte and vessel/vascular area) is shown and normalized to the value of control mice. (**c**) Retinal vascular leakage with FITC-dextran (70 kD) is evaluated and relative retinal vascular leakage (%) of diabetic retina is shown normalized to the value of control mice. The sample size for each group is indicated on the bar graph. The bar graphs represent mean±S.E.M.; *P<0.01 by Student's *t-*test

To determine whether retinal astrocyte coverage to retinal vessel is attenuated in diabetic mice retina, we performed immunohistochemistry in retinal flat mounts of diabetic mice. Retinal vascular coverage by glial fibrillary acidic protein (GFAP)-labeled astrocyte was evaluated in 3 weeks of STZ-induced diabetes and age-matched non-diabetic control mice. Intriguingly, focal loss of astrocyte coverage to retinal vessel was observed in diabetic retina (Figure 1a). The relative astrocyte coverage to retinal vessel (42.0±6.4%) was significantly decreased in the diabetic retina compared to that of the control retina (100.0±5.4%, *P*<0.001; Figure 1b). The retinal vascular leakage was also evaluated with fluorescein isothiocyanate (FITC)-dextran. The retinal vascular leakage (196.5±7.1%) was significantly increased in the diabetic retina compared to that of the control retina (100.0±16.0%, *P*<0.001; Figure 1c). In early diabetic mice, retinal astrocyte loss occurred with increase in retinal vascular leakage.

### Inhibition of Ang2 reduces astrocyte loss and vascular leakage in early diabetic retina

**Figure 2:**
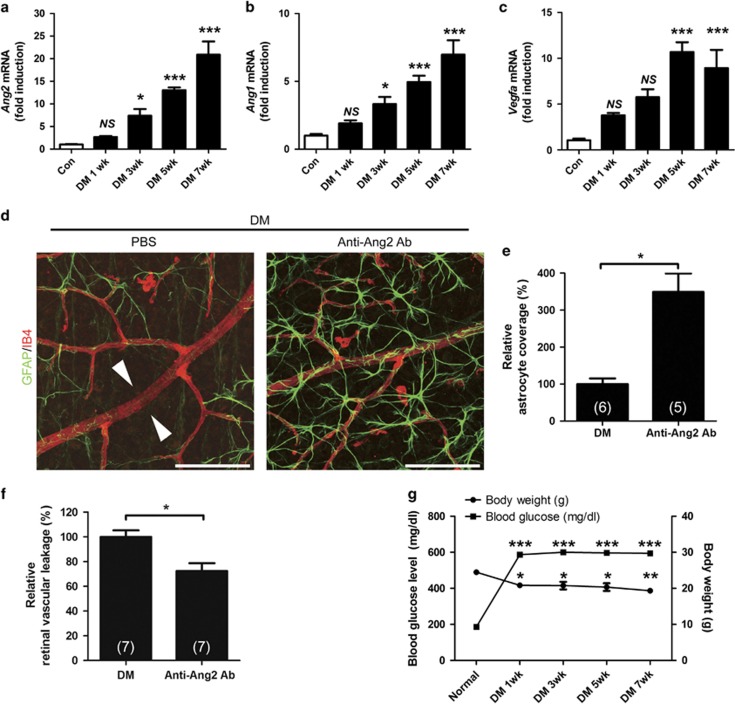
Inhibition of Ang2 reduces the astrocyte loss and vascular leakage in early diabetic retina. (**a**–**c**) Retinal mRNA was determined in 1, 3, 5, and 7 weeks from streptozotocin-induced diabetic mice (DM) and control mice (Con) retinas by qPCR, and normalized to *Rn18s* mRNA. (**a**) *Ang2* mRNA. (**b**) *Ang1* mRNA. (**c**) *Vegfa* mRNA. (**d**–**f**) Anti-Ang2-neutralizing antibody (Anti-Ang2 Ab, 1 *μ*g) or PBS was intravitreally injected to 2-week-old DM. Retinal astrocyte and retinal vascular leakage were evaluated 1 week after the injection in 3-week-old DM. (**d**) Focal astrocyte loss shown in diabetic retina (PBS) is rescued in anti-Ang2 Ab-injected diabetic mice (original magnification × 400; scale bar, 100 *μ*m). White arrowheads indicate loss of astrocytes on diabetic retinal vessels. (**e**) Relative astrocyte coverage (% Anti-Ang2 Ab) is calculated by colocalized area per IB4^+^ vascular area and normalized to the value of control mice. (**f**) Relative retinal vascular leakage (% Anti-Ang2 Ab) with FITC-dextran is shown normalized to the value of control mice. (**g**) Body weight and blood glucose level of the STZ-induced diabetic and non-diabetic control mice at 7 weeks. The sample size for each group is indicated on the bar graph. The bar graphs represent mean±S.E.M.; *P<0.05, **P<0.01, and ***P<0.001 by ANOVA and Student's *t-*test

To determine which growth factor is linked to increased vascular leakage and astrocyte loss in early diabetic retina, we performed ELISA for retinal vascular endothelial growth factor (VEGF) and Ang1/2. Retinal Ang2 level was increased at 1 and 3 weeks after diabetic induction (from 88.9±1.3 pg/mg to 98.11±2.2 and 102.7±4.6 pg/mg, respectively, *P*=0.018 by one-way ANOVA; Supplementary Figure S1a). With *post hoc* test, retinal Ang2 at 3 weeks was significantly increased compared to control (*P*=0.017; Supplementary Figure S1a). However, retinal VEGF and Ang1 levels were not significantly changed up to 3 weeks after diabetic induction (Supplementary Figures S1b and c). In qPCR analysis of diabetic retina (up to 7 weeks after diabetic induction), *Ang2* and *Ang1* mRNAs were significantly elevated after 3 weeks (Figures 2a and b). However, *Vegfa* mRNA was significantly increased after 5 weeks. (Figure 2c). The physical and metabolic parameters of the mice used in ELISA and qPCR were shown in Figure 2g. To explore the possibility that the increased vascular leakage was due to the astrocyte loss by Ang2, we injected Ang2-blocking antibody in diabetic mice at 2 weeks after STZ injection. The retina of anti-Ang2-blocking antibody-injected mice showed increased astrocyte coverage (349.3±49.8%) compared to the loss of astrocyte coverage in PBS-injected diabetic mice (100.0±15.2%, *P*<0.05; Figures 2d and e). In addition, anti-Ang2-blocking antibody attenuated vascular leakage (72.4±6.3%) compared to the vascular leakage in PBS-injected diabetic retina (100.0±5.3%, *P*<0.05; Figure 2f). These findings suggested that astrocyte loss by Ang2 was associated with retinal vascular leakage and it could contribute to retinal vascular leakage in the STZ-diabetic mice.

### Ang2 synergistically induces astrocyte apoptosis with high glucose

**Figure 3:**
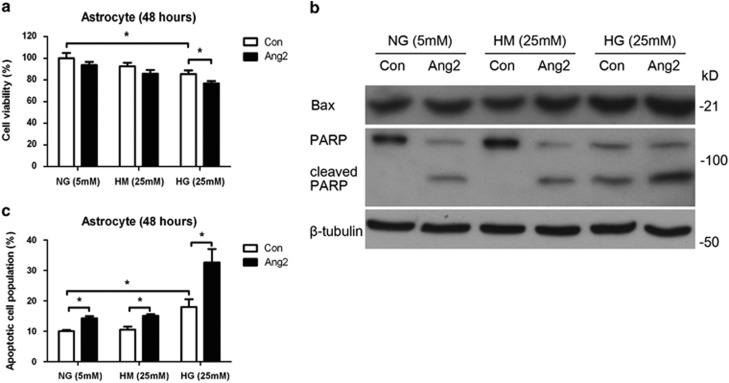
Ang2 synergistically induces astrocyte apoptosis with high glucose (HG; 25 mM glucose). The effects of Ang2 on the cell viability and apoptosis of astrocyte under HG condition were determined. Astrocytes were incubated for 48 h with Ang2 (300 ng/ml) under HG and compared with control (Con). (**a**) Cell viability was assessed by MTT assay. Ang2 aggravated HG-induced cell death in astrocyte. (**b**) Western blot analysis for Bax and cleaved PARP were performed on lysates obtained from astrocytes with the same experimental setting. *β*-Tubulin was used as a loading control. (**c**) Astrocytes were stained with annexin-V FITC and PI, and analyzed by flow cytometry. Cell apoptosis was expressed as the percentage of apoptotic cells in total cell populations. HG induced astrocyte apoptosis and Ang2 aggravated that apoptosis. The bar graphs represent mean±S.E.M. of three independent experiments. HM, high mannitol (5 mM glucose+20 mM mannitol); NG, normal glucose (5 mM glucose). *P<0.05 by Student's *t-*test

To determine the effect of Ang2 on cell viability of astrocyte *in vitro*, we performed 3-(4,5-dimethylthiazol-2-yl)-2,5-diphenyltetrazolium bromide (MTT) assay and fluorescence-activated cell sorting (FACS) analysis. Ang2 leads to pericyte death under high-glucose conditions.^16^ Similarly, it aggravated astrocyte cell death under high glucose (76.8±2.2% from 85.5±3.3%, *P*<0.05; Figure 3a). Next, we examined whether astrocyte cell death is mediated by apoptotic pathway. Indeed, Ang2 induced apoptosis pathway with increase of Bax and cleaved poly(ADP-ribose) polymerase (PARP) under high glucose (Figure 3b). Then, astrocyte apoptosis was assessed by annexin-V/propidium iodide (PI) FACS analysis. Ang2 increases apoptotic cell population regardless of the glucose level. Of importance, high-glucose-induced astrocyte apoptosis was significantly aggravated by Ang2 (32.6±4.4% from 18.0±2.6%, *P*<0.05; Figure 3c). These data suggested that Ang2 induced astrocyte apoptosis especially under high-glucose conditions.

### Ang2 induces astrocyte apoptosis via GSK-3*β*/*β*-catenin pathway under high glucose

**Figure 4:**
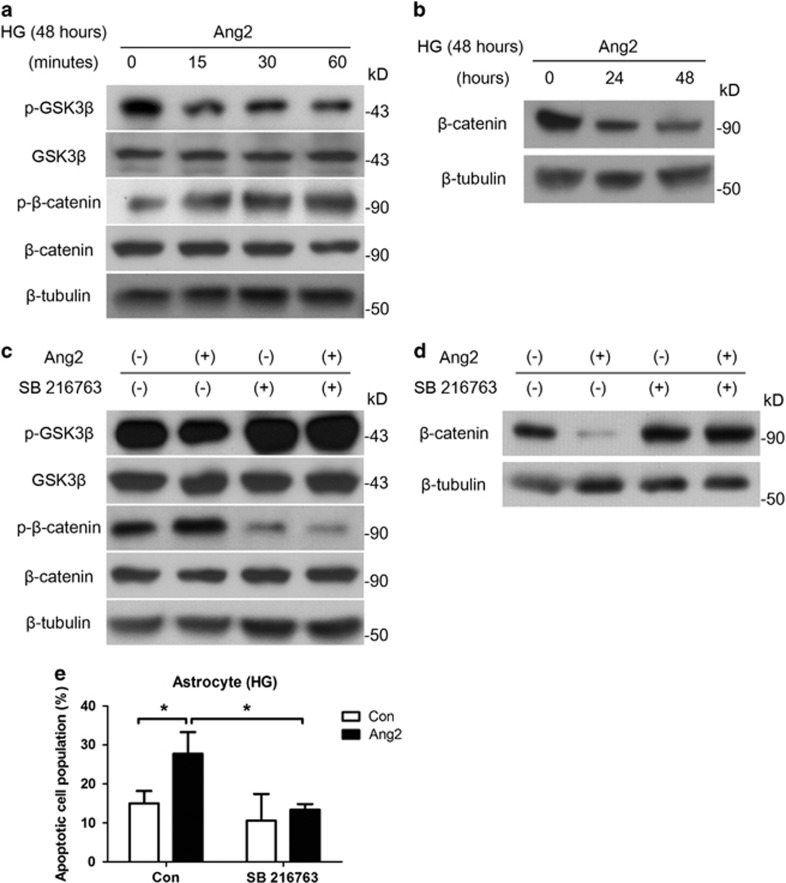
Ang2 induces astrocyte apoptosis via GSK-3*β*/*β*-catenin pathway under high glucose (HG; 25 mM glucose). Although astrocytes were incubated for 48 h under HG, Ang2 (300 ng/ml) was treated for the indicated time period. (**a**) Western blot analysis for phospho-GSK-3*β* (Ser9) and phosphor-*β*-catenin (Thr41/Ser45) were performed on lysates obtained from astrocytes treated with Ang2 for 15, 30, and 60 min under HG (48 h). (**b**) Western blot analysis for *β*-catenin was performed on lysates obtained from astrocytes treated with Ang2 for 24 and 48 h under total 48 h of HG. (**c**) After 48 h of HG incubation, SB216763 (GSK-3*β* inhibitor, 10 *μ*M) was pretreated for 1 h. Then, Ang2 was treated for 30 min. Western blot analysis was performed for phospho-GSK-3*β* (Ser9) and phosphor-*β*-catenin (Thr41/Ser45). (**d** and **e**) Astrocytes were incubated under HG with either Ang2 or SB 216764 for 48 h. (**d**) Western blot analysis for *β*-catenin was performed. (**e**) Apoptotic cell counts were assessed by FACS analysis. The bar graph represents mean±S.E.M. of three independent experiments. *P<0.05 by Student's *t-*test. Data represent three independent experiments. *β*-Tubulin was used as a loading control

We aimed to identify the mechanism that mediates Ang2-induced astrocyte apoptosis under high glucose. Although astrocytes were incubated for 48 h under high glucose, Ang2 (300 ng/ml) was treated for the indicated time period. We found that Ang2 induced GSK-3*β* activation by de-phosphorylation of GSK-3*β* (Ser9) and *β*-catenin phosphorylation (Figure 4a), which in turn led to *β*-catenin degradation under high glucose (Figure 4b). Then, to determine the role of the GSK-3*β*/*β*-catenin pathway for the observed Ang2-mediated astrocyte apoptosis, we treated astrocyte with GSK-3*β* inhibitor (SB216763, 10 *μ*M, 1 h pretreatment). Inhibition of GSK-3*β* reduced Ang2-induced *β*-catenin phosphorylation at 30 min (Figure 4c), and subsequent *β*-catenin degradation at 48 h (Figure 4d). As expected, inhibition of GSK-3*β* effectively attenuated Ang2-induced astrocyte apoptosis under high glucose (13.4±0.8% from 27.8±3.2%, *P*<0.05; Figure 4e). These data suggested that Ang2 induced astrocyte apoptosis via GSK-3*β*/*β*-catenin pathway under high-glucose conditions.

### High glucose increases integrin *α*v*β*5 as an Ang2 receptor in astrocyte

**Figure 5:**
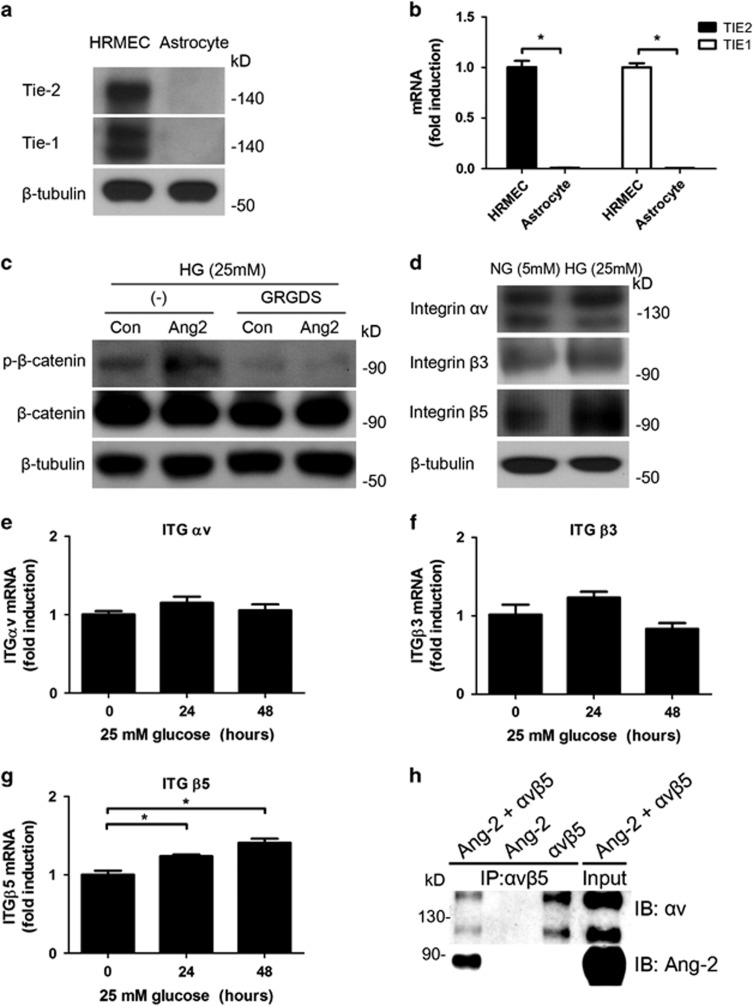
High glucose increases integrin *α*v*β*5 as an Ang2 receptor in astrocyte. (**a**) Western blot for Tie-2 and Tie-1 expression were performed on lysates obtained from HRMECs and astrocytes. (**b**) *TIE2* and *TIE1* mRNA transcriptions were assessed by quantitative RT-PCR. *TIE2* and *TIE1* mRNA levels were normalized to *β-actin* mRNA and reported as fold induction compared to HRMECs. *P<0.001 by Student's *t-*test. (**c**) Western blot analysis for phosphor-*β*-catenin (Thr41/Ser45) and *β*-catenin were performed on lysates obtained from astrocytes treated with Ang2 (300 ng/ml) or GRGDS (0.5 mg/ml) for 30 min after 48 h of 25 mM high-glucose (HG) incubation. (**d**) Western blot analysis for integrin *α*v, *β*3, and *β*5 were performed on lysates obtained from astrocytes under HG for 48 h. *β*-Tubulin was used as a loading control. Data represent three independent experiments. *ITGαv* (**e**), *ITGβ3* (**f**), and *ITGβ5* (**g**) mRNA transcriptions were assessed and normalized to *β-actin* mRNA by quantitative RT-PCR. All mRNA levels were reported as fold induction compared to normal glucose (NG, 5 mM). *P<0.05 by Student's *t-*test. (**h**) After incubation of Ang2 (500 ng), integrin *α*v*β*5 (500 ng) and integrin *α*v*β*5 antibody (2 *μ*g), the immune complexes were co-immunoprecipitated to show direct binding of Ang2 to integrin *α*v*β*5. Then, they were analyzed for Ang2 and integrin *α*v. Same amounts of recombinant Ang2 and integrin *α*3*β*1 were used as an input. Con, control

We aimed to reveal which receptor is responsible for mediating Ang2-induced apoptosis in astrocyte. To determine whether Tie-2 receptor, well-known receptor for Ang2, is related with Ang2-induced astrocyte apoptosis, western blot analysis (Figure 5a) and real-time PCR (Figure 5b) for Tie-2 and Tie-1 were performed on lysates obtained from human retinal microvascular endothelial cells (HRMECs) and astrocyte. As shown in previous studies,^16, 21^ astrocyte did not express Tie-2 or Tie-1 while HRMECs expressed Tie-2 and Tie-1. Compared to HRMECs with Tie-2 expression, astrocyte did not express Tie-2 in FACS analysis (data now shown). Next, to determine whether integrin may serve as receptors for Ang2 in astrocyte apoptosis under high glucose, we incubated astrocyte under high glucose for 30 min with Ang2 and H-Gly-Arg-Gly-Asp-Ser-OH (GRGDS) peptides. GRGDS (0.5 mg/ml) attenuated Ang2-induced *β*-catenin phosphorylation (Figure 5c). This result showed that integrin signaling was involved in Ang2-induced *β*-catenin phosphorylation.

Next, to determine whether the high glucose changes the expression pattern of integrin, we performed quantitative RT-PCR and western blot studies for *α*v, *β*3, and *β*5, which are known to be expressed in astrocyte.^21^ Astrocyte mainly expressed *α*v and *β*5 integrin, and intriguingly, high-glucose conditions increased *β*5-integrin expression (Figure 5d). Although high glucose did not increase *ITGαv* (Figure 5e), *ITGβ3* (Figure 5f) mRNA levels for 48 h, it significantly increased *ITGβ5* mRNA level (*P*=0.002 by one-way ANOVA, 24 h: 1.24±0.04-fold; *P*<0.05 and 48 h: 1.41±0.00-fold, *P*=0.002 by *post hoc* test, respectively; Figure 5g). On the other hand, high glucose decreased *ITGβ8* mRNA (0.72±0.01-fold for 48 h, *P*<0.01; Supplementary Figure S2). In addition, we aimed to show direct binding of Ang2 to integrin *α*v*β*5 as *α*v is an exclusive partner for *β*5. Indeed, Ang2 was shown to directly bind to integrin *α*v*β*5 in co-immunoprecipitation assay (Figure 5h). These data suggested that high-glucose conditions increased integrin *α*v*β*5 as a receptor for Ang2 in astrocyte.

### Ang2-induced astrocyte apoptosis is inhibited by suppression of *α*v*β*5 integrin

**Figure 6:**
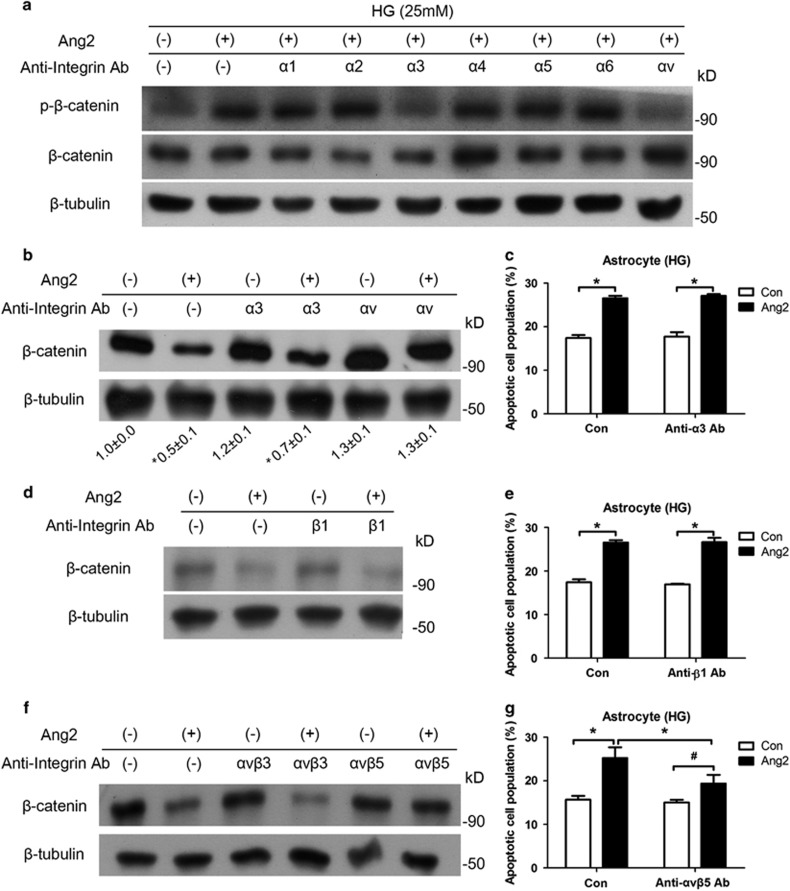
Ang2-induced astrocyte apoptosis is inhibited by suppression of *α*v*β*5 integrin. (**a**) Western blot analyses for phosphor-*β*-catenin (Thr41/Ser45) and *β*-catenin were performed on lysates obtained from astrocytes treated with Ang2 (300 ng/ml) or various integrin-blocking antibodies (10 *μ*g/ml, *α*1, *α*2, *α*3, *α*4, *α*5, *α*6, and *α*v) under 25 mM high glucose (HG) for 30 min. (**b** and **c**) Astrocytes were incubated under HG with Ang2 and anti-integrin *α*3 or *α*v antibodies for 48 h. (**b**) Western blot analysis for *β*-catenin was performed (ImageJ quantification; *N*=3, mean±S.E.M.). (**c**) Astrocyte apoptosis with anti-integrin *α*3 antibody was analyzed by FACS and expressed as the percentage of apoptotic cells in total cell populations. (**d** and **e**) Astrocytes were incubated under HG with Ang2 and anti-integrin *β*1 antibody (10 *μ*g/ml) for 48 h. (**d**) Western blot analysis for *β*-catenin was performed. *β*-Tubulin was used as a loading control. Data represent three independent experiments. (**e**) Astrocyte apoptosis with anti-integrin *β*1 antibody was analyzed by FACS and expressed as the percentage of apoptotic cells in total cell populations. (**f** and **g**) Astrocytes were incubated under HG with Ang2 and anti-integrin *α*v*β*3 or *α*v*β*5 antibodies (10 *μ*g/ml) for 48 h. (**f**) Western blot analysis for *β*-catenin was performed. *β*-Tubulin was used as a loading control. Data represent three independent experiments. (**g**) Astrocyte apoptosis with anti-integrin *α*v*β*5 antibody was analyzed by FACS and expressed as the percentage of apoptotic cells in total cell populations. *P<0.05, ^#^*P*>0.05 compared with Ang2 treatment by Student's *t-*test

To determine which integrin subunit is responsible for Ang2-induced astrocyte apoptosis, we screened the integrin *α* subunits (*α*1-6 and *α*v). Integrin *α*3- and *α*v-blocking antibodies attenuated Ang2-induced *β*-catenin phosphorylation (Figure 6a). To further explore the precise integrin complex, we tested integrin *α*3-, *α*v-, and *β*1-blocking antibodies. Only integrin *α*v-blocking antibody significantly prevented Ang2-induced *β*-catenin degradation (Figures 6b and d). Indeed, integrin *α*3- and *β*1-blocking antibodies did not affect Ang2-induced astrocyte apoptosis (Figures 6c and e). Thus, we could narrow down the candidate to *α*v and its counterpart subunits *β*3 and *β*5. Same experimental procedures were conducted with *α*v*β*3- and *α*v*β*5-integrin-blocking antibodies, and Ang2-induced *β*-catenin degradation was inhibited only by blocking *α*v*β*5 integrin (Figure 6f). Suppression of *α*v*β*5 integrin effectively decreased Ang2-induced astrocyte apoptosis (19.3±1.4% from 25.2±1.2%, *P*<0.05; Figure 6g).

### Inhibition of *α*v*β*5 integrin reduces astrocyte loss in diabetic retina

**Figure 7:**
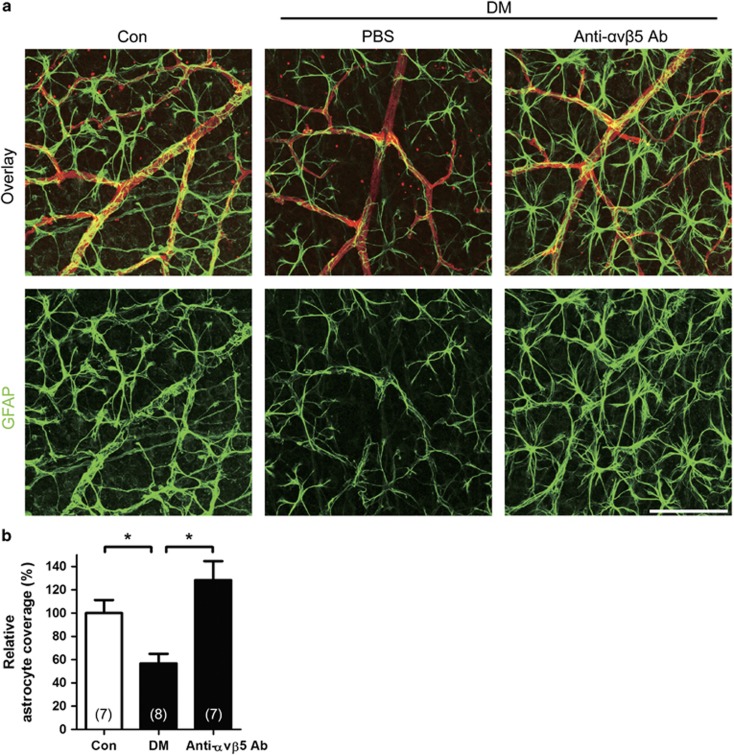
Inhibition of *α*v*β*5 integrin reduces astrocyte loss in STZ-induced diabetic mice (DM). Anti-*α*v*β*5-integrin-neutralizing antibody (Anti-*α*v*β*5 Ab, 1 *μ*g) or PBS was intravitreally injected to 2-week-old DM. Retinal astrocyte was evaluated 1 week after the injection in 3-week-old DM. (**a**) Focal astrocyte loss shown in diabetic retina (PBS) is rescued in anti-*α*v*β*5 Ab-injected DM (original magnification × 400; scale bar, 100 *μ*m). (**b**) Relative astrocyte coverage (%) is calculated by colocalized area of astrocyte and vessel per IB4^+^ vascular area and normalized to the value of control mice. The sample size for each group is indicated in the bar graph. The bar graph represents mean±S.E.M.; *P<0.001 by Student's *t-*test

On the basis of *in vitro* experiments, we next intravitreally injected 1 *μ*g of anti-*α*v*β*5-integrin antibodies to diabetic mice 2 weeks after STZ induction. One week after intravitreal injection, the isolated retinas were stained with GFAP for astrocyte evaluation (Figure 7a). *In vivo*, intravitreal injection of anti-*α*v*β*5-integrin antibody effectively reduced astrocyte loss in diabetic retina (128.3±16.4%) compared to the loss in PBS-injected diabetic mice (53.1±8.6%, *P*=0.003; Figure 7b). These *in vivo* data suggested that astrocyte loss occurred via *α*v*β*5 integrin in diabetic retina. In summary, we demonstrated that Ang2 induced astrocyte apoptosis via *α*v*β*5 integrin in diabetic retina.

## Discussion

In this study, we demonstrated that vascular leakage occurred in the diabetic retina as early as 3 weeks after diabetic induction in mice. Interestingly, the vascular leakage in the early DR was accompanied with loss of astrocytic endfeet. In terms of the neurovascular unit, astrocyte loss could contribute to vascular leakage in the retina. Of importance, our results showed that inhibition of Ang2 effectively attenuated both astrocyte loss and vascular leakage in the early DR. Recently, we demonstrated that Ang2 induced pericyte loss via integrin signaling in DR.^16^ Although pericyte loss is an early characteristic change in DR, vascular leakage can occur even before the pericyte loss in diabetic mice.^5^ Thus, we postulated that astrocyte, as a component of neurovascular unit in retina along endothelial cell and pericyte, could play an important role in early vascular leakage in diabetic retina.

The early glial change in the diabetic retina has been described in diabetic animal models.^6, 7, 15, 22,23,24^ Retinal neuroglial abnormalities are observed predominantly in the inner retina before the characteristic vascular lesions are observed.^7^ In diabetic retina, it has been observed that Müller cells acquire strong GFAP immunoreactivity throughout the retina (reactive gliosis) later than astrocyte change, whereas astrocyte progressively decreased in number and GFAP immunoreactivity.^6, 7, 15^ However, there was no study to investigate the role of astrocyte loss in diabetic retinal vascular leakage in terms of neurovascular unit. Importantly, the molecular mechanism of astrocyte loss in diabetes has not been elucidated yet. Thus, we approached with this concept and also suggested that Ang2-induced astrocyte apoptosis via integrin signaling as the precise molecular mechanism of astrocyte loss in DR.

Ang2 induced astrocyte loss by apoptotic mechanism. We focused on the integrin as a receptor for Ang2 in astrocyte, since astrocyte lacked the expression of Tie-2, a known receptor for Ang2.^20, 21^ Indeed, Ang2 could bind to integrin *α*3*β*1, *α*v*β*5, and *α*5*β*1.^16, 20^ Interestingly, integrin *α*3- or *β*1-blocking antibodies did not affect Ang2-induced astrocyte apoptosis, whereas they mediated Ang2-induced pericyte apoptosis.^16^ Because we showed integrin *β*1 was not responsible to Ang2-induced astrocyte apoptosis (Figures 6d and e), we could rule out many integrin *α* subtypes and focus on the integrin *α*v complexes. Only *α*v*β*5 integrin, not *α*v*β*3 integrin, could mediate Ang2-induced astrocyte apoptosis. Although *α*v integrin did not change significantly, high glucose changed integrin subtype of astrocyte from *β*8 to *β*5, which was responsible for Ang2-mediated astrocyte apoptosis. Because *α*v integrin is the only *α* subtype for the integrin heterodimer with *β*5 subtype,^25, 26^ the increase of *β*5 subtype implied the increase of *α*v*β*5 integrin. In addition, astrocyte mainly expressed *α*v and *β*5 integrins as previously reported.^21, 27^ We confirmed that inhibition of *α*v*β*5 integrin effectively prevented Ang2-induced astrocyte loss *in vitro* and *in vivo*. Thus, integrin *α*v*β*5 could be a therapeutic target in Ang2-induced astrocyte apoptosis.

Regarding to the molecular mechanism of Ang2-induced astrocyte apoptosis, we demonstrated that Ang2 activated GSK-3*β* and subsequently induced *β*-catenin phosphorylation and degradation. In addition, we showed that GSK-3*β*-specific inhibitor blocked Ang2-induced astrocyte apoptosis. Inhibition of GSK-3*β* has been suggested as a therapeutic target in many disease including diabetes and Alzheimer's disease.^28, 29^ Thus, we suggest that it could also be a therapeutic target in diabetic retina. Furthermore, nuclear translocation of *β*-catenin is critical to the activation of TCF/*β*-catenin target genes, including *survivin*, *cyclin D1*, and *c-myc*. The target genes, *β-catenin*, *survivin*, *c-Myc*, and *Cyclin D1* mRNA, significantly decreased in astrocytes treated with Ang2 under high glucose (data not shown). Because survivin is a member of the inhibitor of apoptosis family of protein, decrease of *β*-catenin can lead to cellular apoptosis.^30^

The limitation of our study is the use of single-STZ-diabetic model. Previously, the most commonly used mouse models with diabetes are induced by chemical toxins such as STZ or alloxan.^31^ This approach replicates some of the early features of DR and has the advantage that the onset of diabetes can be defined as the time of STZ injection. However, toxin-induced diabetes in mice has been less successful because of strain-dependent resistance to STZ.^32^ In addition, DR features could be compounded by inflammatory effect of STZ. Despite the drawbacks of STZ-diabetic mice model, we showed high enough glucose levels during experimental periods (Figure 2g). Recently, the availability of the Ins2Akita mouse line enables to use diabetic mice without STZ or toxins.^33^ Interestingly, this Ins2Akita mice also showed decreased GFAP immunoreactivity in astrocytes similar to our observation although there was no explanation about molecular mechanism. Thus, further study using Ins2Akita mice about Ang2 and its role in astrocyte loss via *α*v*β*5-integrin signaling could strengthen our study.

In this study, we provided the evidence for the precise mechanism on the Ang2-induced astrocyte apoptosis under high glucose. These molecular mechanisms gave us the insight for the therapeutic target of astrocyte loss in diabetic retina. In addition, high glucose increases Ang2 in HRMECs^16, 34^ and the diabetic retina.^34, 35^ Thus, inhibition of each steps of Ang2/integrin *α*v*β*5/GSK-3*β* axis could be a potential therapeutic target to treat vascular leakage in early DR.

In conclusion, we demonstrated that Ang2 induced astrocyte loss and vascular leakage in early DR. Ang2 induced astrocyte apoptosis via GSK-3*β*/*β*-catenin pathway under high glucose. High glucose increased *α*v*β*5 in astrocyte. Furthermore, we showed that Ang2-induced astrocyte apoptosis was effectively attenuated by blocking *α*v*β*5 integrin both *in vitro* and *in vivo*. Taken together, Ang2 induced astrocyte apoptosis via *α*v*β*5-integrin signaling contributed to the vascular leakage in the early diabetic retina. We suggest that glycemic control or blocking Ang2/integrin signaling could be a potential therapeutic target to prevent astrocyte loss and vascular leakage in early DR.

## Materials and Methods

### Cell cultures

HRMECs (Applied Cell Biology Research Institute, Kirkland, WA, USA) and human brain astrocytes (Applied Cell Biology Research Institute) were maintained in M199 medium and DMEM with 20% FBS containing growth factors (Hyclone, Logan, UT, USA), respectively. All cells were cultured at 37 °C in an incubator with a humidified atmosphere of 5% CO_2_.

### Reagents and antibodies

Recombinant human Ang2, human integrin *α*v*β*5, phycoerythrin (PE)-conjugated anti-endothelial-specific receptor tyrosine kinase 2 (Tie-2) mouse IgG antibody, mouse Ang2 ELISA kit, mouse VEGF ELISA kit, human phospho-kinase array kit, anti-integrin *α*v*β*3, and anti-integrin *α*v*β*5 (clone P5H9, MAB2528) neutralizing antibodies were purchased from R&D systems (Minneapolis, MN, USA). Mouse Ang1 ELISA kit was purchased from MyBioSource (San Diego, CA, USA). Anti-Bax antibody was purchased from Epitomics (Burlingame, CA, USA). Anti-phospho GSK-3*β* (Ser9), anti-phospho *β*-catenin, anti-*β*-catenin, and anti-PARP antibody were purchased from Cell Signaling Technology (Beverly, MA, USA). Anti-Tie-1 and peroxidase-conjugated secondary antibodies were purchased from Santa Cruz Biotechnology (Dallas, TX, USA). Anti-Tie-2 antibody, GRGDS peptide, and SB216763 were purchased from Millipore (Billerica, MA, USA). MTT (3-(4,5-dimethylthiazol-2-yl)-2,5-diphenyltetrazolium bromide), STZ, and FITC-dextran (70 kD) were purchased from Sigma-Aldrich (St. Louis, MO, USA). FITC-conjugated annexin-V/PI assay kit were purchased from BD Biosciences (Franklin Lakes, NJ, USA). Anti-GFAP, anti-integrin *α*v, *β*1, *β*3, *β*5, and anti-Ang2 antibodies were purchased from Abcam (Cambridge, MA, USA). Anti-mouse Ang2-blocking antibody (Angy-2-1) was purchased from Adipogen (San Diego, CA, USA). Anti-integrin *α*3 (clone P1B5, MAB1952), anti-integrin *β*1 (clone 6S6, MAB2253), and *α*-integrin-blocking and IHC kit (*α*1-6, v) were purchased from Millipore and used for functional blocking.

### Animals

All animal experiments in this study were in strict agreement with the Association for Research in Vision and Ophthalmology Statement for the Use of Animals in Ophthalmic and Vision Research and the guidelines of the Seoul National University Animal Care and Use Committee. Six-week-old, pathogen-free male C57BL/6J mice were purchased from Central Lab. Animal Inc. (Seoul, Korea). Diabetes was induced by a single intraperitoneal injection of freshly prepared STZ at a concentration of 180 mg/kg body weight in 10 mM citrate buffer (pH 4.5) as previously reported.^16^ Age-matched controls received citrate buffer only. Mice with blood glucose levels >300 mg/dl 4 days after STZ injection were deemed diabetic. Diabetic and non-diabetic mice were killed 3 weeks after diabetes induction, and eyes were collected under deep anesthesia and immediately frozen at −80 °C for ELISA, or fixed in 4% paraformaldehyde for retinal flat mount. Glucose levels (Accu-Chek, Roche, Indianapolis, IN, USA) and body weight were monitored consecutively.

### ELISA

Two retinas from mice were pooled (*N*=6 per each group). Samples including retina, vitreous, and lens were homogenized and lysed with brief sonication in 500 *μ*l RIPA buffer with a complete protease inhibitor cocktail (Roche). After centrifugation at 12 000 r.p.m. for 20 min, supernatants were collected. Ang1, Ang2, and VEGF levels were measured using ELISA kits according to the manufactures' instructions. The protein concentration was measured using a BCA protein assay kit (Pierce, Rockford, IL, USA). Ang1 (ng), Ang2 (pg), and VEGF levels (pg) were standardized to the retina protein (mg).

### Quantitative and qualitative assessment of the retinal vascular leakage

To determine vascular leakage, 3-week-old STZ-induced diabetic mice were used. Deeply anesthetized mice were intravenously injected with a FITC-dextran dissolved in PBS. After 1 h of perfusion, the eyes were enucleated and fixed in 4% paraformaldehyde for 1 h. The retinas were dissected in 2 × PBS, flat-mounted and viewed by a fluorescent microscope (Eclipse 90i, Nikon, Tokyo, Japan) at a magnification of × 40 and × 100.

To analyze vascular leakage quantitatively, the four representative sites of leakage at the mid-peripheral retina (0.5 *μ*m x 0.5 *μ*m) per each mice were selected (*N*=6–7). The mid-peripheral retina was designated as the mid-one-third of the retina from optic nerve head to ciliary body. The images were imported into ImageJ (1.47v, NIH, Bethesda, MD, USA); thereby, images were adjusted for color threshold based on the automatic isodata algorithm, and the interested area with FITC was marked with red. Then, the area of red was measured. The data were expressed as vascular leakage (%), which was normalized to those of control mice.

### Quantification of retinal vascular coverage by astrocyte

To determine the morphology and quantitative vascular coverage of retinal astrocyte, retinal flat mounts were immunostained against GFAP for astrocyte and IB4 for vessel. The retinas were observed by a confocal microscope (Leica TCS STED, Leica Microsystems, Wetzlar, Germany). Briefly, the eyes were enucleated and fixed in 4% paraformaldehyde for 1 h. The retinas were dissected out in 2 × PBS and incubated in 100% methanol overnight at −20 °C. Then, the retinas were blocked in Perm/Block solution (0.3% Triton-X and 0.2% BSA in PBS) for 1 h at room temperature. Then, the retinas were incubated with anti-GFAP antibody (1 : 100) and Alexa Fluor 594-conjugated anti-IB4 antibody (1 : 100) in Perm/Block solution overnight at 4 °C. Then, the retinas were repeatedly washed with PBS and incubated with secondary antibody (1 : 200) for 1 h at room temperature. After washed with PBS, the retinas were mounted with Fluoromount aqueous mounting medium (Sigma-Aldrich). The representative site of astrocyte loss at the mid-peripheral retina per each mice was selected (*N*=6–8). The morphology of astrocyte was analyzed with maximal projection images of *z*-stack (magnification × 400). For the analysis of the vascular coverage by astrocyte, the colocalized area of astrocyte and retinal vessel was divided by the area of retinal vessel (astrocyte coverage=colocalized area/vascular area). The colocalized area was calculated by using the Leica Application Suite Advanced Fluorescence (LAS AF) software (Leica Microsystems; threshold and background values are 30 and 20%, respectively). The images were imported to Imaris software (Ver.7.6.5, Bitplane, Belfast, UK), and analyzed for the vascular area. Then, the vascular coverage by astrocyte was normalized to that of control (relative astrocyte coverage % Supplementary Figure 3).

### Intravitreal injection of anti-Ang2 and anti-integrin-neutralizing antibodies

One microliter containing 1 *μ*g of anti-Ang2-neutralizing antibody and 1 *μ*g of anti-integrin *α*v*β*5 antibody (clone P5H9) was injected intravitreally under deep anesthesia at 2 weeks after diabetes induction with STZ. Sterile PBS was injected for control. After 7 days of injection, retinas were subjected to the retinal flat mount for the vascular leakage and/or astrocyte analyses.

### Cell viability assay

In all experiments, 2.5 × 10^4^ cells were seeded into 96-well plates. After 24 h, cells were treated with Ang2 (300 ng/ml) under normal glucose (5 mM glucose), high glucose (25 mM glucose), and high mannitol (5 mM glucose plus 20 mM mannitol) as an osmotic control for 48 h. Cell viability was determined by MTT assay according to the manufacturer's instructions. Three independent experiments were performed for each experimental condition.

### FACS analysis

In order to evaluate the apoptosis, 5 × 10^5^ cells were treated with Ang2 (300 ng/ml) under normal glucose, high mannitol, and high glucose at 37 °C for 48 h. To determine the effect of integrin blocking, anti-integrin-blocking antibodies (10 *μ*g/ml) were treated 1 h before the addition of Ang2. The cells were collected and washed two times in PBS. Cells were stained with FITC annexin-V and PI for 15 min, and analyzed by flow cytometry. Annexin V-positive/PI-negative cells were determined to apoptotic.

### Quantitative RT-PCR

Total RNA was collected and isolated from cells using RNeasy Plus Mini kit (Qiagen, Valencia, CA, USA). cDNAs were prepared from RNAs (1 *μ*g) using 2.5 *μ*M oligo-dT primers, 1 mM dNTPs, and MuLV reverse transcriptase. Quantitative real-time PCR (qPCR) assays were performed in qPCR master mix for SYBR Green PCR master mix (Applied Biosystems, Life technologies, Gaithersburg, MD, USA) using 7900HT real-time PCR (Applied Biosystems). qPCR conditions were set as follows: (i) 95 °C for 10 min and (ii) 50 cycles of 5 s at 95 °C and 20 s at 60 °C. qPCR was performed using the primers in Supplementary Table S1. A mean quantity was calculated from triplicate qPCR for each sample and it was normalized to the control gene.

Total RNA was isolated from retina using TRI Reagent (Molecular Research Center, Cincinnati, OH, USA) according to the manufacturer's instructions. cDNA was prepared with High Capacity RNA-to-cDNA kit (Life Technologies). Real-time PCR was performed with StepOnePlus Real-Time PCR System (Life Technologies) with TaqMan Fast Advanced Master Mix (Life Technologies) and specific Gene Expression Assays (cat. no. 4331182; Life Technologies). Product IDs of Gene Expression Assays for genes are as follows: for Angpt1, Mm00456503_m1; for Angpt2, Mm00545822_m1; for Vegfa, Mm00437306_m1; and for Rn18s, Mm03928990_g1. All procedures were performed in accordance with the MIQE guidelines.

### Immunoprecipitation and immunoblotting

For immunoprecipitation, Ang2 (500 ng) and integrin *α*v*β*5 (500 ng) were precleared with G-Sepharose beads (Millipore) at 4 °C for 1 h. Then, the complex and anti-integrin *α*v*β*5 antibody (2 *μ*g) were incubated with G-Sepharose beads at 4 °C overnight. The immune complexes were collected by centrifugation and washed with buffer three times. For immunoblotting, cells were collected and lysed in RIPA buffer with a protease inhibitor cocktail. Protein lysates were resolved by SDS polyacrylamide gel and transferred onto nitrocellulose membrane. The membranes were incubated with primary antibodies (1 : 1000) overnight at 4 °C and secondary antibodies (1 : 10 000) for 1 h at room temperature. The membranes were incubated with enhanced chemiluminescent substrate (Pierce) and exposed to a film or LAS 4000 (GE Healthcare, Piscataway, NJ, USA).

### Statistical analysis

Statistical analyses were performed using the standard two-tailed Student's *t-*test for two groups, and one-way ANOVA and *post hoc* test with Bonferroni's multiple comparison for multiple groups, respectively (SPSS Statistics 20.0, IBM, Armonk, NY, USA). *P<0.05 was considered statistically significant. Quantitative data and figures are given as mean±S.E.M.

## Abbreviations

Ang2: angiopoietin 2
BBB: blood-brain barrier
BRB: blood-retinal barrier
DR: diabetic retinopathy
FACS: fluorescence-activated cell sorting
FITC: fluorescein isothiocyanate
GFAP: glial fibrillary acidic protein
GRGDS: H-Gly-Arg-Gly-Asp-Ser-OH
HRMECs: human retinal microvascular endothelial cells
MTT: 3-(4,5-dimethylthiazol-2-yl)-2,5-diphenyltetrazolium bromide
PARP: poly(ADP-ribose) polymerase
PI: propidium iodide
STZ: streptozotocin
VEGF: vascular endothelial growth factor.
