# Supplementary figures and images for: Angiopoietin 2 induces astrocyte apoptosis via αvβ5-integrin signaling in diabetic retinopathy
Source: Cell Death Dis. 2016 Feb 18;7(2):e2101–. doi: 10.1038/cddis.2015.347 (PMC5399183; doi:10.1038/cddis.2015.347)

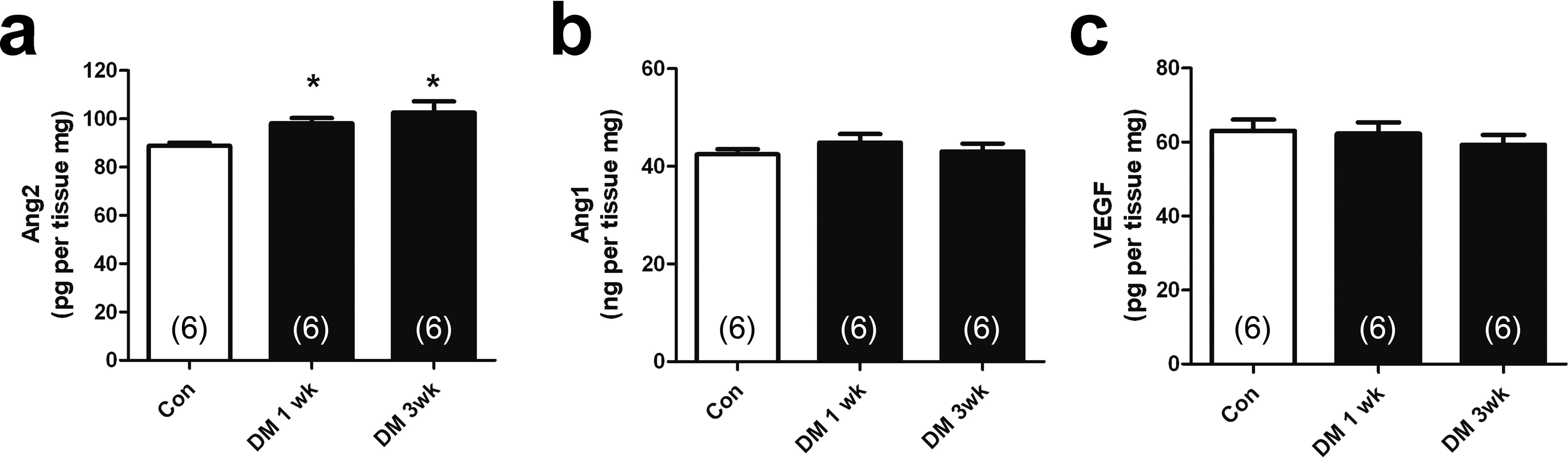

Supplement: Supplementary Figure 1 [file cddis2015347x1.tif]

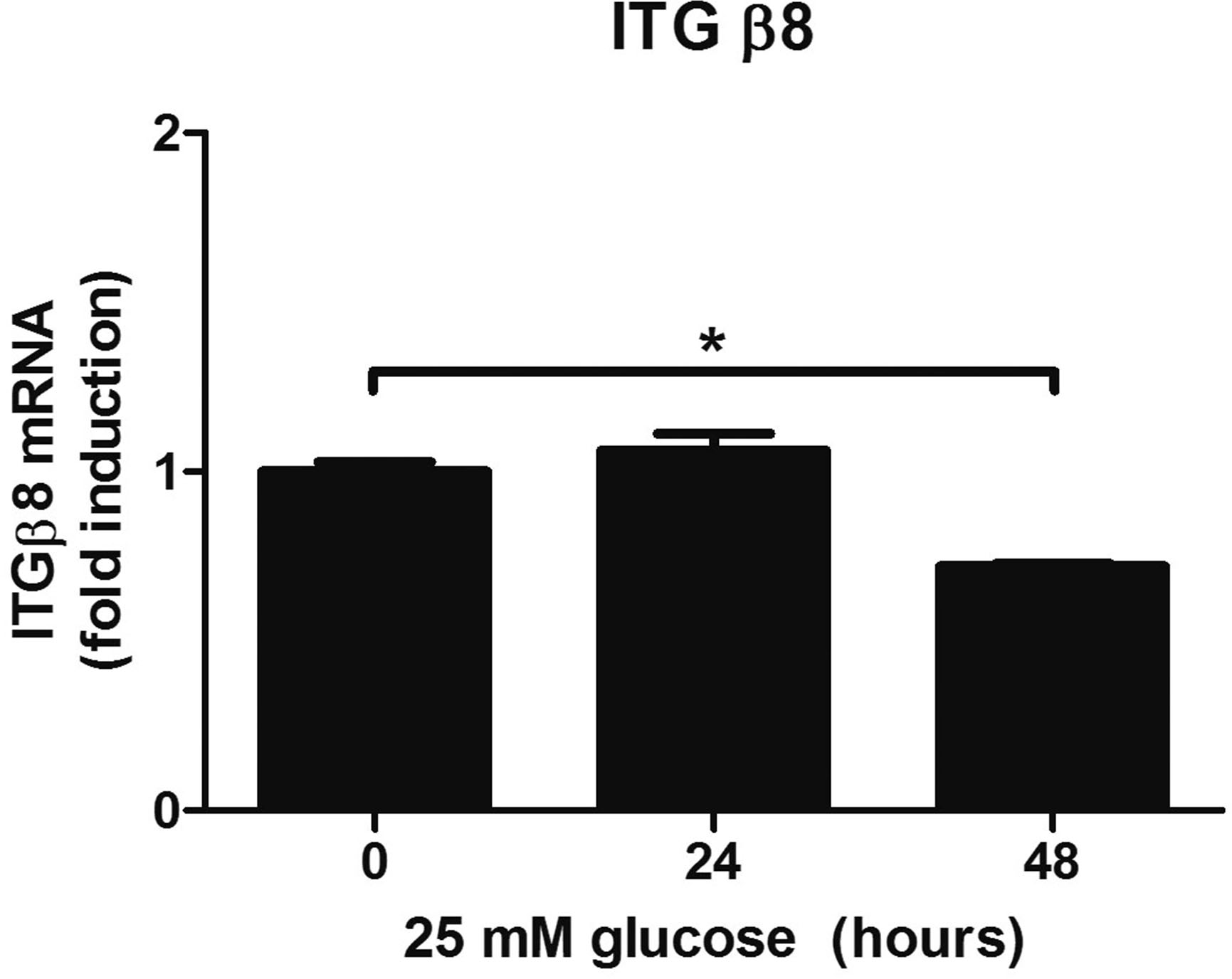

Supplement: Supplementary Figure 2 [file cddis2015347x2.tif]

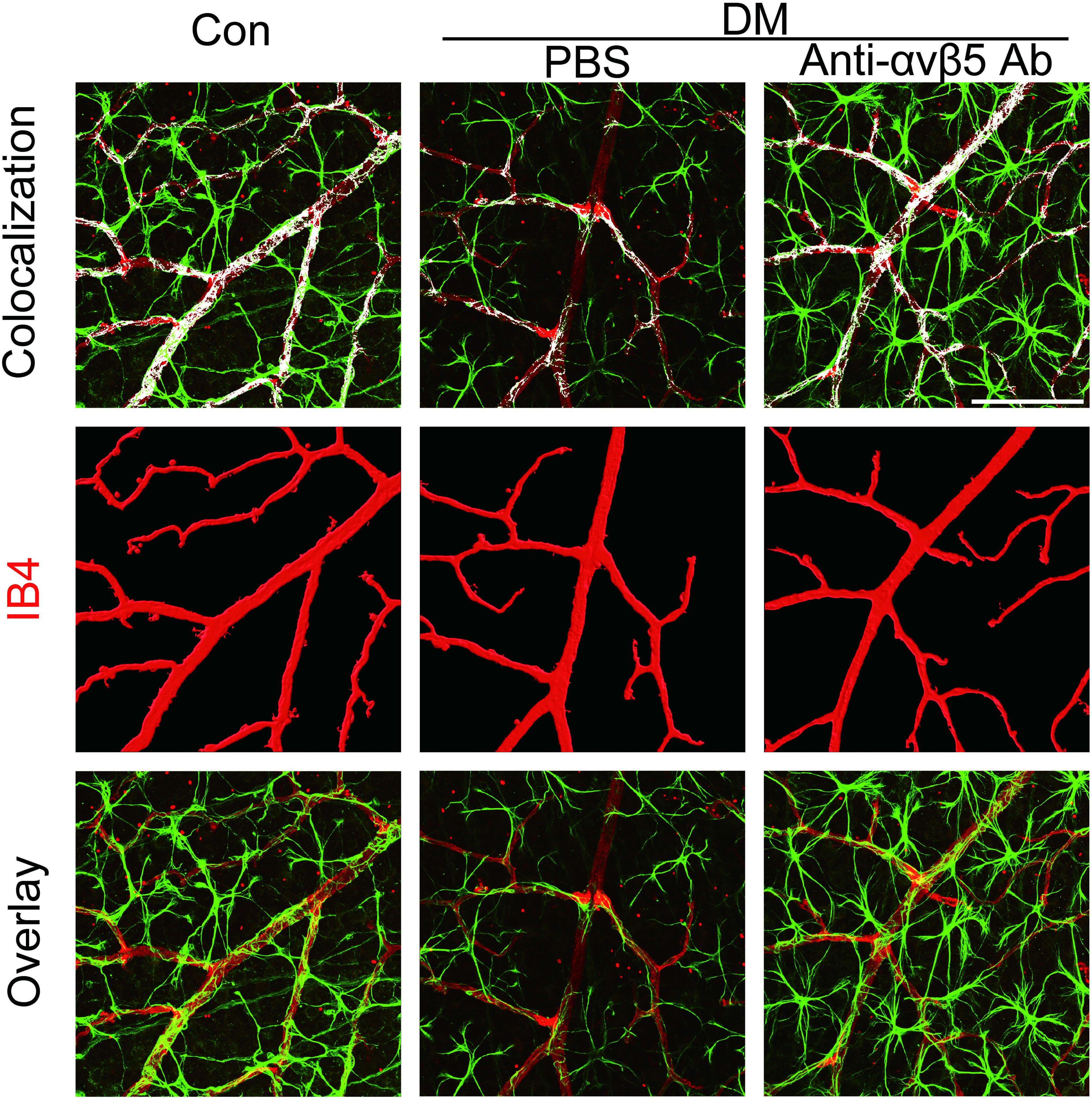

Supplement: Supplementary Figure 3 [file cddis2015347x3.tif]
