# Supplementary material for: Angiopoietin 2 induces astrocyte apoptosis via αvβ5-integrin signaling in diabetic retinopathy
Source: Cell Death Dis. 2016 Feb 18;7(2):e2101–. doi: 10.1038/cddis.2015.347 (PMC5399183; doi:10.1038/cddis.2015.347)
Supplement: Supplementary Figure Legends [file cddis2015347x4.doc]

| Supplementary Table S1. qPCR primer sequences | |
| --- | --- |
| **Primers** | **Sequence** |
| Human TIE1 (forward) | 5’ AGAACCTAGCCTCCAAGATT 3’ |
| Human TIE1 (reverse) | 5’ ACTGTAGTTCAGGGACTCAA 3’ |
| Human TIE2 (forward) | 5’ GCTTGCTCCTTTCTGGAACTGT 3’ |
| Human TIE2 (reverse) | 5’ CGCCACCCAGAGGCAAT 3’ |
| Human ITGαv(forward) | 5’ AATCTTCCAATTGAGGATATCAC 3’ |
| Human ITGαv (reverse) | 5’ AAAACAGCCAGTAGCAACAAT 3’ |
| Human ITGβ3 (forward) | 5’ CCGTGACGAGATTGAGTCA 3’ |
| Human ITGβ3 (reverse) | 5’ AGGATGGACTTTCCACTAGAA 3’ |
| Human ITGβ5 (forward) | 5’ GGAGCCAGAGTGTGGAAACA 3’ |
| Human ITGβ5 (reverse) | 5’ GAAACTTTGCAAACTCCCTC 3’ |
| Human ITGβ8 (forward) | 5’ AATTTGGTAGTGGAAGCCTATC 3’ |
| Human ITGβ8 (reverse) | 5’ GTCACGTTTCTGCATCCTTC 3’ |
| Human β-ACTIN (forward) | 5’ GCCGCCAGCTCACCAT 3’ |
| Human β-ACTIN (reverse) | 5’ TCGATGGGGTACTTCAGGGT 3’ |
