# Supplementary material for: Angiopoietin 2 induces astrocyte apoptosis via αvβ5-integrin signaling in diabetic retinopathy
Source: Cell Death Dis. 2016 Feb 18;7(2):e2101–. doi: 10.1038/cddis.2015.347 (PMC5399183; doi:10.1038/cddis.2015.347)
Supplement: Supplementary Table 1 [file cddis2015347x5.doc]

**Legends to Supplementary Figures**

**Supplementary Figure S1: Ang2 increases in early diabetic retina.** (a-c) Retinal protein level was determined in 1 and 3 weeks from streptozotocin-induced diabetic mice (DM) and control mice (Con) retinas by ELISA, and normalized to tissue. (a) Ang2 (pg/mg) expression increased in DM retinas. (b) Ang1 (ng/mg) expression did not change significantly. (c) VEGF (pg/mg) expression did not change significantly. The sample size for each group is indicated on the bar graph. The bar graphs represent mean ± SEM, **P* < 0.05 by Student’s *t-*test.

**Supplementary Figure S2. High glucose decreases integrin β8 in astrocyte.**

Astrocytes were incubated under 25 mM glucose (HG) for 48 h. *ITGβ8* mRNA transcriptions was assessed and normalized to *β-ACTIN* mRNA by quantitative RT-PCR. The result was reported as fold induction compared to normal glucose (5 mM glucose). **P* < 0.05 by Student t test.

**Supplementary Figure S3. The quantification method of relative astrocyte coverage to retinal vessel.**

The maximal projection images (z-stack size: 16 images, original magnification ×400; scale bar = 100 μm) were imported to Imaris software (Bitplane). Colocalized area of glial fibrillary acidic protein (GFAP)+ astrocyte (green) and IB4+ vessel (red) is shown with white pixels. Surface area of IB4+ vessel (red) were marked and calculated. For the analysis of the vascular coverage by astrocyte, the colocalized area of astrocyte and retinal vessel was divided by the area of retinal vessel (astrocyte coverage = colocalized area / vascular area). Then, the vascular coverage by astrocyte was normalized to that of control (Relative astrocyte coverage %).
